# Introduction to the special issue in commemoration of Olaf Breidbach

**DOI:** 10.1007/s12064-016-0235-9

**Published:** 2016-09-05

**Authors:** Jürgen Jost

**Affiliations:** 1 Max Planck Institute for Mathematics in the Sciences, Inselstr. 22, 04103 Leipzig, Germany; 2 Santa Fe Institute, 1399 Hyde Park Rd., Santa Fe, NM 87501 USA

This is the first of two special issues that Theory in Biosciences is dedicating to the memory of Olaf Breidbach, its long time editor who had transformed the traditional “Biologisches Zentralblatt” into the modern “Theory in Biosciences”, a journal dedicated to the theoretical, conceptual and historical aspects of biology in its broadest sense. Olaf Breidbach had passed away prematurely in July 2014 at the age of 56. Several obituaries have been published, like (Bach 2014a, b; Jost 2014b), including one in this journal (Jost 2014a).

The articles in this issue, as well as in the second one which will appear in 2017, have been contributed by his colleages and friends. The wide range of topics treated in those contributions reflects the immense scope of the intellectual interests of Olaf Breidbach as well as the respect and admiration that he has enjoyed among scientists from many different scientific disciplines. In fact, biology has only been one amongst a great variety of research topics of Olaf Breidbach. He was not only an accomplished biologist with highly original, deep, and penetrating contributions, but also one of the most eminent and prolific historians of science, an insightful philosopher, a theoretician of art, a complex systems thinker with unorthodox views and novel perspectives, and in general a widely respected intellectual, as described in detail in the obituaries cited above. This journal, due to its nature and scope, can only publish articles of biological relevance, but the two special issues dedicated to his memory can hopefully, nevertheless, provide a good indication of the impressive intellectual depth and range of Olaf Breidbach.

José F. Fontanari and Francisco A. Rodrigues in their contribution “Influence of network topology on cooperative problem-solving systems” investigate how the connectivity structure of social networks and the resulting information transmission in such networks affect the ability of social groups to find optima in fitness landscapes and how they can avoid becoming trapped in local maxima that are not global fitness peaks.

Luiz R. R. Faria, Elaine Della Giustina Soares, Eduardo do Carmo and Paulo Murilo Castro de Oliveira in “Diploid male dynamics under different numbers of sexual alleles and male dispersal abilities” investigate the spreading of diploid males in a haplodiploid system of sexual determination. Under a single locus complementary sex-determination system, the homozygotes at that locus become diploid males. When, as assumed in their model, such diploid males are infertile, but cannot be distinguished by females from fertile haploid males, then, because these diploid males are disadvantageous for the population, this may even lead to the extinction of that population. The authors study the corresponding dynamics in terms of male flight abilities and number of sexual alleles.

Parth Pratim Pandey and Sanjay Jain in “Analytic derivation of bacterial growth laws from a simple model of intracellular chemical dynamics” investigate the growth laws of bacterial cells in steady-state cultures, and they can derive the empirical growth laws in a simple mathematical model. In their model, the cellular growth rate can be explicitly determined. The relevant parameters turn out to be the number of amino acid residues per enzyme and per ribosome. From this, many consequences can be derived, for instance a characterization of resource allocation at the maximal growth rate.

Wolfgang Banzhaf et al. address a key issue in artificial life and the theory of evolution, the issue of open-endedness of evolution, in their article “Defining and simulating open-ended novelty: requirements, guidelines, and challenges”. The general question is whether biological or social or artificial evolution can become even more complex or whether it will necessarily eventually saturate and not be able to produce forever novelty. The authors make this issue more precise by defining several types of novelty, variation, innovation, and emergence. On this basis, they can discuss the difficulties and identify the challenges of this concept in depth and provide a meta-model for open-endedness that can capture aspects common to the different fields of biological evolution, socio-economic systems and computer simulations.

Alessandro Minelli develops the theory of “Scaffolded biology”. The interest in this concept emerged from the dissatisfaction with standard dichotomies like organism vs. environment, nature vs. nurture, genetic vs. epigenetic, and from the desire to better conceptualize the interactions between those complementary entities. Building upon various approaches in the literature, Minelli defines a scaffold as any resource utilized by a biological system without using it in metabolism or incorporating it in some other way. The concept thus is abstract enough to capture the utilization of genetically related or unrelated species members, organisms from other species, external material as in niches, etc. Minelli investigates the hybrid relationship between a scaffold and a scaffolded system, focussing on such relations between individual organisms or their parts.

Finally, in “The evolution of the plant genome-to-morphology auxin circuit”, Ulrich Kutschera and Karl J. Niklas discuss and analyze the general principles of developmental evolutionary biology from both a historical perspective and in the light of recent research paradigms. They start from the seminal work of Ernst Haeckel who had first proposed an evolutionary approach to development, culminating in his biogenetic law. In modern terms, the issue is the map from the genome to the morphology. Kutschera and Niklas discuss a concrete example, the plant hormone auxin (indole-3-acetic acid, IAA). Endogenous IAA is involved in many developmental processes in plants. It is clear that the pathways by which IAA is synthesized must be developmentally regulated, but many details are still unclear, even in the model organism *Arabidopsis thaliana*, and further progress requires the integration of molecular, biochemical, physiological and organismal knowledge with evolutionary insight.

Thus, the articles in this volume present a wide panorama of evolution, from concrete case studies in zoology (the population dynamics in the presence of infertile diploid males) and in botany (the evolutionary genome-to-morphology IAA circuit) to mathematical models (on the influence of the topology of cooperative networks on the problem-solving ability as well as about bacterial growth laws) and computer simulations (of networks and of artificial evolution) to conceptual issues (the principle of scaffolding and the question of the open-endedness of evolution), and they also include the historical perspective, focussing on the role of Ernst Haeckel in developing an evolutionary perspective on ontogenetic development.

The latter is most appropriate, as Olaf Breidbach had been the director of the Ernst-Haeckel-Haus in Jena for twenty years and did fundamental work on Ernst Haeckel, both from the perspective of the history of science and from conceptual thinking in evolution. In particular, he edited many of Haeckel’s works, see for instance (Breidbach and Haeckel 1996, 1998, 2006, 2012). Among his many contributions to the theories of Ernst Haeckel in journals and edited volumes, let me just mention (Breidbach 2002, 2006). Olaf Breidbach was also interested in the theory of evolution in general, and we have been collaborating on such issues for many years (Breidbach and Jost 2004; Schindler et al. 2013), and in Breidbach (1995), he discussed the role of development as a foundation of evolutionary theory. In fact, already in his early work on the neuroanatomy and -physiology of beetles, the evolutionary perspective was a fundamental ingredient, see for instance (Kutsch and Breidbach 1994) and his contributions with W. Kutsch in their edited volume (Breidbach and Kutsch 1995).

Certainly, Olaf Breidbach would have enjoyed all the contributions in this volume.
